# Slow Recovery of Excitability Increases Ventricular Fibrillation Risk as Identified by Emulation

**DOI:** 10.3389/fphys.2018.01114

**Published:** 2018-08-28

**Authors:** Brodie A. Lawson, Kevin Burrage, Pamela Burrage, Christopher C. Drovandi, Alfonso Bueno-Orovio

**Affiliations:** ^1^ARC Centre of Excellence for Mathematical and Statistical Frontiers, School of Mathematical Sciences, Queensland University of Technology, Brisbane, QLD, Australia; ^2^Department of Computer Science, University of Oxford, Oxford, United Kingdom

**Keywords:** rotors, arrhythmias (cardiac), fibrillation, excitability, refractoriness, emulation, machine learning, Gaussian process regression

## Abstract

**Purpose:** Rotor stability and meandering are key mechanisms determining and sustaining cardiac fibrillation, with important implications for anti-arrhythmic drug development. However, little is yet known on how rotor dynamics are modulated by variability in cellular electrophysiology, particularly on kinetic properties of ion channel recovery.

**Methods:** We propose a novel emulation approach, based on Gaussian process regression augmented with machine learning, for data enrichment, automatic detection, classification, and analysis of re-entrant biomarkers in cardiac tissue. More than 5,000 monodomain simulations of long-lasting arrhythmic episodes with Fenton-Karma ionic dynamics, further enriched by emulation to 80 million electrophysiological scenarios, were conducted to investigate the role of variability in ion channel densities and kinetics in modulating rotor-driven arrhythmic behavior.

**Results:** Our methods predicted the class of excitation behavior with classification accuracy up to 96%, and emulation effectively predicted frequency, stability, and spatial biomarkers of functional re-entry. We demonstrate that the excitation wavelength interpretation of re-entrant behavior hides critical information about rotor persistence and devolution into fibrillation. In particular, whereas action potential duration directly modulates rotor frequency and meandering, critical windows of excitability are identified as the main determinants of breakup. Further novel electrophysiological insights of particular relevance for ventricular arrhythmias arise from our multivariate analysis, including the role of incomplete activation of slow inward currents in mediating tissue rate-dependence and dispersion of repolarization, and the emergence of slow recovery of excitability as a significant promoter of this mechanism of dispersion and increased arrhythmic risk.

**Conclusions:** Our results mechanistically explain pro-arrhythmic effects of class Ic anti-arrhythmics in the ventricles despite their established role in the pharmacological management of atrial fibrillation. This is mediated by their slow recovery of excitability mode of action, promoting incomplete activation of slow inward currents and therefore increased dispersion of repolarization, given the larger influence of these currents in modulating the action potential in the ventricles compared to the atria. These results exemplify the potential of emulation techniques in elucidating novel mechanisms of arrhythmia and further application to cardiac electrophysiology.

## 1. Introduction

Self-sustaining patterns of aberrant excitation in the heart, re-entries, are the cause of dangerously accelerated heartrates (tachycardia) and complete losses of synchronized action (fibrillation) (Wit and Cranefield, 1978). Re-entrant circuits often form around unexcitable anatomical features such as veins, with the properties of these obstacles then primarily defining the resulting excitation behavior (Gough et al., 1985; Cherry et al., 2007). However, so-called “functional” re-entries can also develop and sustain themselves in unimpeded tissue (Moe and Abildskov, 1959; Allessie et al., 1977), manifesting as spiral waves that are clinically known as rotors (Pandit and Jalife, 2013). The behavior of functional re-entries depends on the electrophysiological properties of the cells composing cardiac tissue, which vary considerably among population members (Sims et al., 2008) and in different regions of the heart (Feng et al., 1998). Understanding the impact of this variability on the generation and persistence of arrhythmic events, and the corresponding implications for success or failure of anti-arrhythmic treatments, is a critical challenge in cardiac electrophysiology (Sobie, 2009; Sarkar et al., 2012; Muszkiewicz et al., 2016; Passini et al., 2017).

Arrhythmic risk is commonly analyzed in terms of “excitation wavelength” (Smeets et al., 1986; Rensma et al., 1988; Tse and Yan, 2017), the product of conduction velocity (CV) and the effective refractory period (ERP) or action potential duration (APD). This determines the minimum length for which re-entrant circuits will sustain electrical activity, and thus increasing wavelength discourages re-entry formation and maintenance (Wiener and Rosenblueth, 1946), and explains the mechanism of action for many anti-arrhythmic drug therapies (Wang et al., 1992). However, anti-arrhythmic drugs that increase wavelength by prolonging APD/ERP may also be pro-arrhythmic (Wolbrette, 2003; Elming et al., 2004), and class I anti-arrhythmic agents that decrease CV (and hence wavelength) are successfully used for rhythm control of atrial fibrillation (Nattel et al., 2003; Kneller et al., 2005). This points to a subtle and still poorly understood interplay between refractoriness and excitability in modulating re-entry. Of particular interest is post-repolarization refractoriness, given confounding evidence that suggests it as both an anti-arrhythmic and pro-arrhythmic mechanism (Kanki et al., 1998; Kirchhof et al., 1998; Muñoz et al., 2007; Coronel et al., 2012; Franz et al., 2014; Cabo, 2015).

Given the expense of experimentation in the heart, and the lack of direct and independent control over properties of interest (such as cell-level electrophysiological properties), *in silico* modeling serves as a critical tool for the understanding of arrhythmia (Zhou et al., 2018). Parameters encoding experimentally elusive properties can be systematically varied by the modeler, and large-scale interrogation of cardiac model output for different values of their parameters has enabled studies of variability (Sobie, 2009; Sarkar et al., 2012; Pathmanathan et al., 2015), parameter inference (Wallman et al., 2014; Johnstone et al., 2016), and the construction of *in silico* populations (Britton et al., 2013; Muszkiewicz et al., 2016; Passini et al., 2017; Lawson et al., 2018). With regard to the complex spatiotemporal dynamics of cardiac rotors, however, previous research has mostly focused on the variation of only one or two parameters at once (Efimov et al., 1995; Fenton and Karma, 1998; Qu et al., 2000; Pandit et al., 2005; Bartocci et al., 2011; Sánchez et al., 2012). Only a small number of studies have considered simultaneous variation in larger numbers of model parameters (Lee et al., 2016; Liberos et al., 2016), but mainly vary ionic current densities and not the kinetic properties of channel recovery. Quantitative understanding of how cell-level electrophysiological properties modulate the complex interactions between refractoriness and excitability when mediated by tissue coupling therefore remains severely lacking.

Emulation is a powerful technique for greatly reducing the computational cost associated with exploration of parameter variability in complex models that are time-intensive to simulate, with a history in climate modeling (Holden and Edwards, 2010) and engineering design (Simpson et al., 2001). In cardiac electrophysiology, while emulators have proved successful in the prediction of electrophysiological properties for single cells (Chang et al., 2015; Johnstone et al., 2016), and for the forward ECG problem (Geneser et al., 2008; Swenson et al., 2011; Johnston et al., 2017), their capabilities remain largely unexplored for the spatiotemporal dynamics of excitation. Only an initial study by the authors did emulate excitation waves in tissue, but in the context of predicting the shapes of steady state wavefronts (Lawson et al., 2017), with no consideration of the far more complex excitation patterns that define arrhythmia.

Here we present an emulation technique, specifically designed for models of cardiac electrophysiology, that significantly reduces the computational cost of exploring variability across many parameters and streamlines the analysis process. We demonstrate and validate our technique by emulating a suite of spatial biomarkers directly related to arrhythmic risk, and apply it to investigate the generation and persistence of rotor-derived tachycardic and fibrillatory excitation behaviors when all important factors modulating tissue excitability and refractoriness are allowed to simultaneously vary. New electrophysiological insights associated with the cardiac excitation wavelength and of particular relevance for ventricular arrhythmias emerge, including the identification of increased risk of wave breakup in response to slower recovery of fast inward channels, and differential effects of decreasing slow inward current or increasing slow outward current despite both changes increasing ERP. Our method extends naturally to biophysically detailed models and realistic heart anatomies.

## 2. Methods

### 2.1. Simulated arrhythmias

Our study focuses on the impacts of variability in cell-level properties on the induction and persistence of re-entry in cardiac tissue. To avoid the impacts of other conflating factors we work in one of the simplest settings for simulating tachycardia and fibrillation. This is a two-dimensional layer of isotropic tissue, allowing for use of the monodomain formulation (Sundnes et al., 2006),

(1) $$\begin{array}{r} \frac{\partial u}{\partial t}=D\nabla^{2}u+I_{\text{ion}}+I_{\text{stim}}. \end{array}$$

Here the membrane potential (expressed in terms of dimensionless variable *u*) spreads by tissue coupling with associated constant *D*, kinetics of cellular excitation and repolarization are encoded in the *I*_ion_ term, and the external supply of stimulus is represented by *I*_stim_. For the description of excitation and repolarization kinetics we selected the reduced Fenton–Karma model (Fenton and Karma, 1998) (hereafter FK model), given its relative speed of simulation and the rich set of re-entrant behaviors that it is capable of replicating (Fenton and Karma, 1998; Fenton et al., 2002). Importantly, the model has been shown to capture the essential tissue-scale properties governing re-entry, being capable of reproducing the re-entrant patterns of more physiologically detailed models for human cardiocytes in the atria (Lombardo et al., 2016), and with slight modifications to also fit action potential (AP) morphology, in the ventricles (Bueno-Orovio et al., 2008). As a reference, we select the parameters for the FK model (Table 1) that correspond to modified Beeler–Reuter dynamics (Beeler and Reuter, 1977; Courtemanche and Winfree, 1991), given the body of work examining the dynamics of waves of excitation in this model (Efimov et al., 1995; Courtemanche, 1996). Full model equations are provided in the Supplementary Material.

**Table 1:** The variable parameters, which control the important properties of cell depolarization and repolarization in response to electrical stimulus.

**Parameter**	**Base value**	**Variability**	**Description**
*g*_*fi*_	3 mS/cm^2^	±30%	Maximum conductance of fast inward (activation) current
*g*_*so*_	0.02 mS/cm^2^	±20%	Maximum conductance of slow outward (repolarization) current for activated cell
*g*_*so*(rest)_	0.12 mS/cm^2^	±30%	Maximum conductance of slow outward (repolarization) current for inactivated cell
*g*_*si*_	0.0223 mS/cm^2^	±20%	Maximum conductance of slow inward (plateau) current
$\tau_{v}^{+}$	3.33 ms	±50%	Time constant for inactivation of fast inward current
$\tau_{v1}^{-}$	1,000 ms	±50%	Initial time constant for reactivation of fast inward current (cell below activation threshold)
$\tau_{v2}^{-}$	19.6 ms	±50%	Secondary time constant for reactivation of fast inward current (cell below activation threshold)
$\tau_{w}^{+}$	667 ms	±50%	Time constant for inactivation of slow inward current
$\tau_{w}^{-}$	11 ms	±50%	Time constant for reactivation of slow inward current
*u*_*c*_	0.13	–	Membrane potential (dimensionless) above which the cell is considered activated
*u*_*si*_	0.85	–	Membrane potential (dimensionless) at which the slow inward current activates
*u*_*v*_	0.055	–	Membrane potential (dimensionless) at which the rate of fast inward channel recovery switches
*k*	10	–	Steepness of the smoothed step function used in the expression for the slow inward current

*Base values for all parameters are those specified by Fenton and Karma for recreating modified Beeler-Reuter dynamics with their model (Fenton and Karma, 1998), except g_fi_ = 3 mS/cm^2^, chosen in order to better explore the variety of rotor trajectories. Grayed out parameters were not varied in the final exploration of the full model, with variation in $\tau_{w}^{-}$ removed after preliminary analysis suggested it was of low importance*.

Simulation software was written in Matlab, using a high-order numerical stencil as described in Bueno-Orovio et al. (2006). Briefly, a second-order Strang splitting (Strang, 1968) is used in time to separate the reaction and diffusion terms of the monodomain equation. The diffusive component is then integrated exactly in Fourier space using a cosine expansion to impose the required non-flux boundary conditions, whereas the reaction term is solved by the modified Euler method (with gating variables integrated by the Rush–Larsen scheme Rush and Larsen, 1978), therefore preserving global second-order time accuracy. All simulations were conducted on two-dimensional tissue layers of 15 × 15 cm in size to allow sufficient space for rotor accommodation, with a diffusion coefficient of *D* = 1 cm^2^/s, constant time step of 0.1 ms, and a space discretization of 512 points in each space direction (~0.03 cm), allowed by the high-order convergence of Fourier spectral methods. The accuracy of the numerical simulations was verified in one-dimensional cables by halving the time and space integration steps. This resulted in <1% change in conduction velocity, even in the low excitability limit.

**Figure 1:**
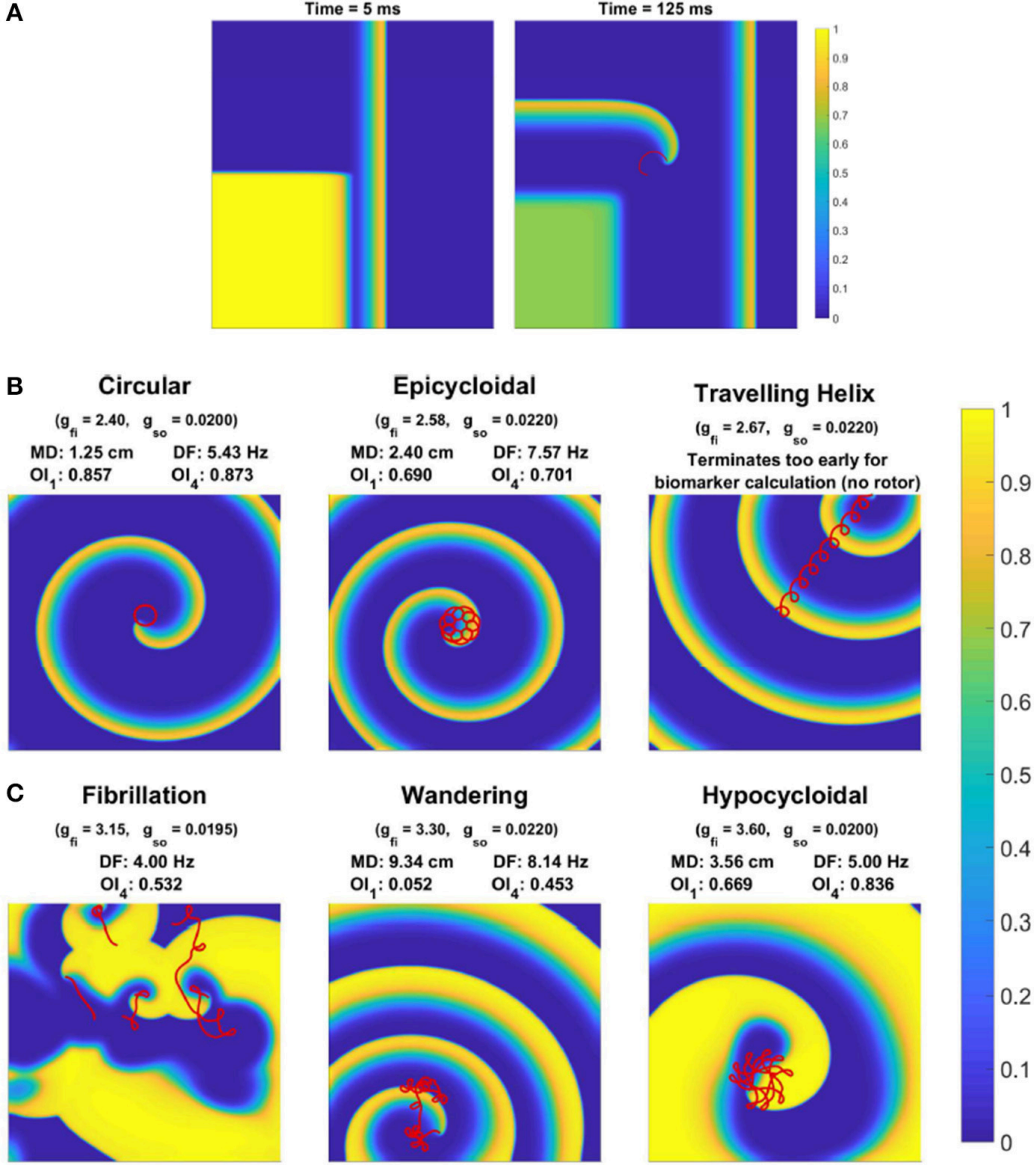
Behavior of rotor-driven re-entries varies significantly in response to electrophysiological variability. **(A)** Induction of a rotor via cross field S1–S2 stimulus, as demonstrated by the membrane potential field 5 and 125 ms after the S2 stimulus. The S1 stimulus creates a planar wave traveling to the right, and the square region stimulated by the S2 stimulus overlaps with the waveback of the initial wave, creating directional block. While excitation propagates upwards, tissue to the right eventually recovers and allows for excitation to curl into this region, forming the beginning of a rotor-driven re-entry. **(B)** Example rotor trajectories and the corresponding excitation field for different choices of some model parameters. All snapshots are taken at 3,000 ms (except for the helix, which terminates early). The last 1,200 ms of rotor tip movement in each case is visualized in red. Values for the spatial biomarkers maximum distance (MD), dominant frequency (DF), and organization indices (OI) are also presented.

Re-entries are generated in our simulations via an S1–S2 stimulation protocol, with the S2 stimulus timed to produce directional block that quickly develops into a phase singularity (rotor tip). The first stimulus acts at one edge of the domain, generating a planar wave. Then, when the waveback of this wave has reached the middle of the domain (as judged by crossing *u* = *u*_crit_ at this location), a second stimulus is provided to one whole quadrant on the wave's back side. With appropriate selection of *u*_crit_ (here *u*_crit_ = 0.05), excitation can propagate in one cardinal direction and not the other, resulting in the generation of a rotor tip in the center of the domain, at the corner of the second stimulated region (Figure 1A). We use the algorithm described by Fenton and Karma (1998) to track the position of rotor tips. Each simulation was run for 8,000 ms after the initial induction of a rotor, but terminated prematurely if all rotor activity died out.

### 2.2. Data generation and biomarker choice

Training and test data used to construct and validate emulators were generated by running the model for different combinations of parameter values across the space of interest, with the design of these computational experiments selected via Latin hypercube sampling (Mckay et al., 1979) by Matlab's *lhsdesign* function. This provides data that is better distributed across the parameter space, improving classifier and emulator performance.

We created two sets of *in silico* data in order to explore the impacts of variability in electrophysiological parameters. Firstly, we introduced variability into two model parameters, *g*_*fi*_ and *g*_*so*_, which are the two current conductances most directly controlling CV and APD, respectively. Such data allowed us to explicitly visualize and thus better demonstrate the effectiveness of our classification and emulation techniques. The second set of *in silico* data allows eight model parameters to vary, including the main current conductances and time constants regulating excitability and repolarization in the model (see Table 1). The reduced dataset was composed of 2,000 simulated electrophysiological scenarios, and the full dataset involved 3,500 due to the higher-dimensional parameter space requiring additional data to be resolved to sufficient accuracy. Variability was accounted as uniform distributions on model parameters (Britton et al., 2013), with ranges of variability as detailed in Table 1. These are consistent with the larger ranges of variability considered in previous studies (Britton et al., 2013; Liberos et al., 2016; Muszkiewicz et al., 2016; Zhou et al., 2016), given the normalized magnitude of the transmembrane potential in the FK model.

Depending on the specific choice of values for these electrophysiological properties, simulations exhibited a range of behaviors. When tissue excitability is too weak, excitation does not propagate and no rotor can form. A rotor also fails to form if cell refractoriness is too long, as the tissue stimulated by the S2 stimulus remains unexcitable and blocks the rotation of the rotor tip, forcing it to collide with the domain boundary and be eliminated. Rotors that are successfully induced can either persist, wander the domain until they are eliminated by a boundary, or devolve into wave breakup corresponding to fibrillation. Figure 1B shows the main rotor behaviors that could be observed. We represented the output of each simulation as a classification of the overall behavior observed, and a set of “spatial biomarkers” that serve as quantitative measures of rotor dynamics and the associated arrhythmic risk. As detailed by Table 2, model output was classified into the four categories suggested above, namely a lack of rotor formation, formation of a stable rotor, formation of a transient rotor, or fibrillatory dynamics. This classification becomes important for constructing predictive emulators, as we explain below.

**Table 2:** The four classifications used to separate different model behaviors, and the conditions used to automatically classify simulation output into each.

**Name**	**Classification conditions**	**Interpretation**
No rotor	No rotor forms, or fails to complete at least one rotation, as judged by detecting APs at probes close to each corner of the domain	Tissue excitability is too weak to propagate signals, or APD is too long for a rotor to rotate after S2 stimulus
Stable rotor	Rotor activity persists for full simulation time and conditions for *Fibrillation* are not met	Persistent rotor-driven tachycardia
Transient rotor	Rotor activity ceases before simulation end, and conditions for *Fibrillation* are not met	Transient rotor-driven tachycardia that self-terminates
Fibrillation	At least five individual rotor tips (phase singularities) that each exist for at least 50 ms form during simulation	Wave breakup that produces a transient or persistent episode of fibrillation

Five phase singularities are required to confidently identify wave breakup, as interactions with the initial condition can occasionally produce multiple rotors even when electrophysiological dynamics do not support wave breakup. The last two classifications are combined in much of the work into a single classification, labeled “Chaotic.”

For biomarkers, we measured steady-state CV and APD prior to the establishment of re-entry, given that they are key properties in determining the cardiac wavelength. These were measured using the S1 stimulus, and so refer to the CV and APD of planar waves in the tissue. We term these the “tissue-level” biomarkers, given that they can be experimentally measured at this scale. For stable rotors, we recorded the greatest distance between any two points on the rotor's trajectory (after it had been given a chance to stabilize), and the organization index derived from the main peak in power spectrum (OI_1_), as measures of the critical size of tissue substrate required to support such a rotor, and the complexity of its trajectory, respectively. Additionally, we created several virtual probes throughout the domain at regular intervals, that measured the time course of the membrane potential at their location. The power spectrum of each of these signals was averaged, and then used to calculate the standard organization index using the main four peaks in power spectrum (OI_4_), as well as the dominant frequency (DF). These provide a measure of the level of chaos present in the rotor dynamics, and the effective rate of induced tachycardia, respectively. Each of these biomarkers, along with more detail regarding their calculation, is provided in Table 3. The values of these biomarkers for different types of rotor behavior are also included in Figure 1B.

**Table 3:** The biomarkers used to characterize simulation behavior, and their interpretations with regard to arrhythmias.

**Biomarker name**	**Measurement**	**Interpretation**
Conduction velocity	Time taken for S1 pulse to cross two markers	Affects inherent rotation rate, wavelength
Action potential duration	Time taken for tissue stimulated by the S1 pulse to return to *u*_crit_ after depolarisation	Affects availability of excitable tissue, wavelength
Maximum distance	Maximum distance between any two points on the rotor tip's trajectory	Critical size required for rotor persistence
Dominant frequency	Location of largest peak in the power spectrum of the signal recorded by virtual probes	Severity of resultant tachycardia
Organization index 1	Proportional contribution of the largest peak in the power spectrum of a single rotor's trajectory to the total power contained in that spectrum	Complexity of a stable rotor's trajectory
Organization index 4	Proportional contribution of the four largest peaks in the power spectrum recorded by virtual probes, to the total power contained in that spectrum	Regularity of rotor circulation

### 2.3. Gaussian process emulation

For emulation, we make use of Gaussian process (GP) regression, as introduced for the emulation of computer models by Sacks et al. (1989). GP regression creates an approximation to the model's response surface for each of the spatial biomarkers, by making use of the generated training data. A good reference for this approach is Rasmussen and Williams (2006). Separate GPs are used for each of the biomarkers, with these GPs characterized by a function μ(**θ**) that defines the mean of the process (in the absence of data) at any point in the parameter space, **θ**, and a covariance function, *k*(**θ**, **θ**′) that defines the covariance between any two points in the parameter space, **θ** and **θ**′. For the basic forms of these functions, we select for the mean function a linear trend, and for the covariance function the Matern-5/2 covariance,

(2) $$\begin{array}{l} k(\theta ,\theta^{\prime })=\sigma^{2}\left(1+\sqrt{5}r+\frac{5r^{2}}{3}\right)\text{exp}\left(-\sqrt{5}r\right)+\sigma_{n}^{2}\delta_{\theta ,\theta^{\prime }},\\ \;r=\sqrt{\sum_{i=1}^{D}\frac{{\left(\theta_{i}-\theta_{i}^{\prime }\right)}^{2}}{l_{i}^{2}}}. \end{array}$$

This function simply dictates that GP predictions at two sets of parameter values **θ** and **θ**′ become more correlated as the two points in parameter space become closer, but with this measure of “closeness” defined such that each dimension in the parameter space can contribute differently (encoded by the choice of *l*_*i*_'s). The values of these *l*_*i*_'s are determined during the training process, and thus the method automatically determines the relative importance of each variable in **θ** toward the output being emulated (known as automatic relevance determination). Here σ controls the overall amount of variance (and covariance) of the process, and σ_*n*_ the noise in the data (with the Kronecker delta used to ensure it contributes to variance at any point, but not covariance). In the case of emulation, the data is output from a deterministic computer simulation and so technically σ_*n*_ = 0, but its inclusion can regularize the process and we here do not assume σ_*n*_ = 0.

Given a set of values for the hyperparameters **l** = (*l*_1_, *l*_2_, …, *l*_*D*_), σ and σ_*n*_, the likelihood of generating the training data with the corresponding GP may be analytically calculated (Rasmussen and Williams, 2006). Thus, we may choose these hyperparameters, along with those specifying the mean function, by maximizing this likelihood using Matlab's built-in function *fitrgp*. Importantly, maximization of this likelihood naturally corresponds to optimizing a balance between data fit and model complexity, discouraging over-fitting (Rasmussen and Williams, 2006).

Once the GP's hyperparameters have been determined, predictions can then be made using simple matrix-matrix and matrix-vector products, if the inverse of the covariance matrix (covariances between all training points) is stored during the training process. Emulator predictions are thus extremely rapid, and the optimization problem involved with training the emulator can be greatly accelerated by the use of derivative information, which can be calculated at very little additional computational cost (Rasmussen and Williams, 2006). The primary cost remains running the simulator in order to generate the initial training data, but this is easily performed in parallel.

### 2.4. Emulator partitioning

GP regression assumes a smooth response of each output to changes in the parameters. Predictions thus suffer when there are critical values of the parameters that cause a sudden change in any of the outputs. Our simulations of rotor dynamics certainly exhibit this property, due to sharp transitions, for example, from simulations that generate a single rotor to simulations that fail to propagate, and from single persistent rotors to wave breakup. It thus becomes necessary to divide the parameter space into separate regions that can then each be emulated by their own Gaussian process. Some previous approaches to this problem in the literature have allowed the boundaries between regions to also be determined during the training process, by defining their locations either using regression trees (Gramacy and Lee, 2008) or Voronoi tesselations (Kim et al., 2005) and then exploring this augmented space via Bayesian sampling techniques. The power of these approaches in detecting where boundaries should be located, without any specification from the user about model behavior (unsupervised learning), comes at the cost of a much longer training process and the risk of determining incorrect boundary locations. In our case, we classify model outputs according to a compact number of general rotor behaviors (Table 2), and hence can take a supervised learning approach. Supervised learning has been used previously in the context of spiral waves in cardiac tissue, but for the separate problem of detecting rotors from image data (Grosu et al., 2009).

With the training data classified into the different behaviors in Table 2, multi-class classification techniques can then be used to predict which category of behavior any given set of parameter values is expected to produce. As long as the regions that correspond to the different behaviors can be well-separated, this leads to distinct regions that can each be assigned their own GP emulator. For a classification model, we use a set of support vector machines (SVM) (Cortes and Vapnik, 1995) that each individually make binary classification predictions, but together form an ensemble that performs multi-class classification. Gaussian kernels (radial basis functions) are used, with hyperparameters selected to optimize performance under five-fold cross validation by Matlab's *fitcecoc*. The “coding design” used to perform multi-class classification is also selected as part of this optimization process, between either one-against-one or one-against-all (see Hsu and Lin, 2002 for a comparison of these and other designs).

### 2.5. Calculation of mean effects

In using our emulator to explore the dynamics of the full model, we calculate the mean effect of each of its parameters upon our spatial biomarkers, a type of global sensitivity analysis (Oakley and O'Hagan, 2004; Chang et al., 2015). These are calculated by averaging over the effects of variability in all other parameters, providing a sense of how a single given parameter affects the output in question among a variable population. Denoting our partitioned emulator Y and using Monte Carlo integration to perform this averaging, the mean effect of a variable θ_*i*_ on a given model output *y* is given by

(3) $$\bar{y}(\theta_{i})\approx \frac{1}{N}\sum_{j=1}^{N}Y(\lambda_{j},\theta_{i}).$$

Here each **λ**_*j*_ is a random realization of all other parameters in **θ**, selected according to their distributions. For the model outputs that can take on null values, we choose to calculate the average over only those sampled points where the emulator predicts non-null values.

We again use LHS to improve the overall coverage over the parameter space in calculating these expectations, and choose a large *N* = 100, 000 to ensure good accuracy in integrating over the effects of variability in the other parameters. With eight variables and 100 points used to represent the functions defined in Equation (3), in total we perform 80 million emulated runs of the two-dimensional FK model. The necessity of emulation in performing such analyses for cardiac electrophysiological models is clear.

## 3. Results

### 3.1. Important features of rotor-driven re-entry are not explained by CV and APD

**Figure 2:**
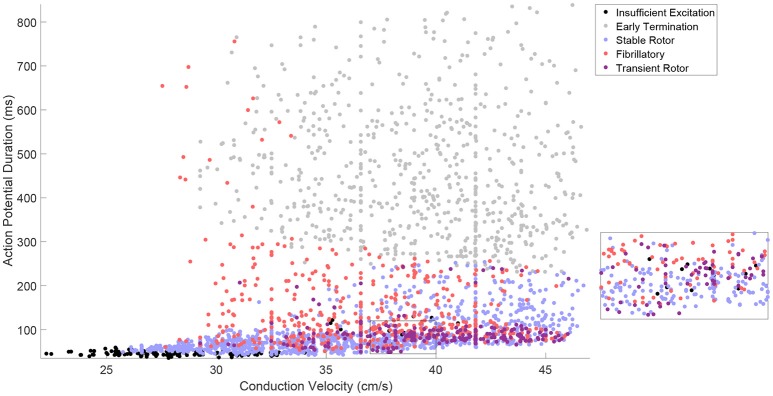
The tissue-level biomarkers CV and APD fail to predict rotor-driven re-entry behavior. The classes of model behavior observed for different values of the tissue-level biomarkers, CV, and APD, with variability in all major model parameters. Classes are assigned according to Table 2, but with the “No Rotor” classification further separated into failure to propagate excitation and early termination (almost always due to APD being too long to successfully induce a rotor with the S1–S2 stimulus) in order to make the visualization more informative. There are regions of the biomarker space where behavior is largely predictable, but on the whole, the class of behavior cannot be predicted from these biomarkers alone and clearly depends on further electrophysiological factors. This is best demonstrated by regions like the one shown in the inset.

Our generated *in silico* data allows us to rigorously explore how well the effects of electrophysiological variability on rotor inducibility and maintenance are captured by the tissue-level biomarkers that can be more readily measured, the steady-state values of CV and APD. The large extent of variability in model parameters resulted in a corresponding amount of variation in CV and APD, with a good spread across the space of these biomarkers, as illustrated in Figure 2. Points in this figure are color-coded according to the type of re-entrant behavior observed in each simulation, revealing a few distinct regions of the biomarker space where behavior is consistent.

Especially predictable are the cases where no re-entry could be induced, suggesting that APD and CV are generally sufficient in themselves for describing the inducibility of re-entry. This includes both the case where CV is very low and excitation fails to properly propagate, and the case where APD is too high and thus the critical length is too long for the spiral wave's tip to successfully rotate before colliding with the simulation boundary. A faster CV decreases the critical APD value beyond which rotors fail to form, because the wavetip travels further while waiting for tissue to recover its excitability. This agrees well with the known importance of wavelength in defining the critical length of re-entrant paths (Wiener and Rosenblueth, 1946; Rensma et al., 1988).

On the other hand, there are large regions of the biomarker space where similar values for the biomarkers result in wholly different re-entrant behaviors, highlighting the importance of finer-scale ionic effects in governing which rotors are likely to persist, annihilate themselves, or exhibit breakup into fibrillation. Transient rotors arise most frequently when conduction and repolarization are both fast, and when APD values are moderate, slow conduction promotes fibrillation while fast conduction promotes stable rotors.

On the whole, these tissue-level biomarkers inform well the critical length required for re-entry establishment, but only poorly the type of re-entrant behavior will result in the case of a spiral wave. Variability in the cell properties themselves, ion channel conductances and time constants, must be considered directly in order to properly understand how this variability manifests in different re-entrant behaviors and the severity of the arrhythmias that result. Furthermore, these findings highlight the importance of looking beyond excitation wavelength when evaluating the anti- or pro-arrhythmic properties of drug treatments.

### 3.2. Partitioned emulation of spatial biomarkers captures the complex dependence of re-entrant behavior on ionic properties

#### 3.2.1. Classifier and emulator predictions

In the case where only two parameters are varied, forward simulation provides enough information about the effects of variability to validate our classification and emulation techniques. This additionally allows for emulator predictions to be visualized, and thus to confirm that our selected spatial biomarkers appropriately capture the important features of rotor-driven re-entry.

**Figure 3:**
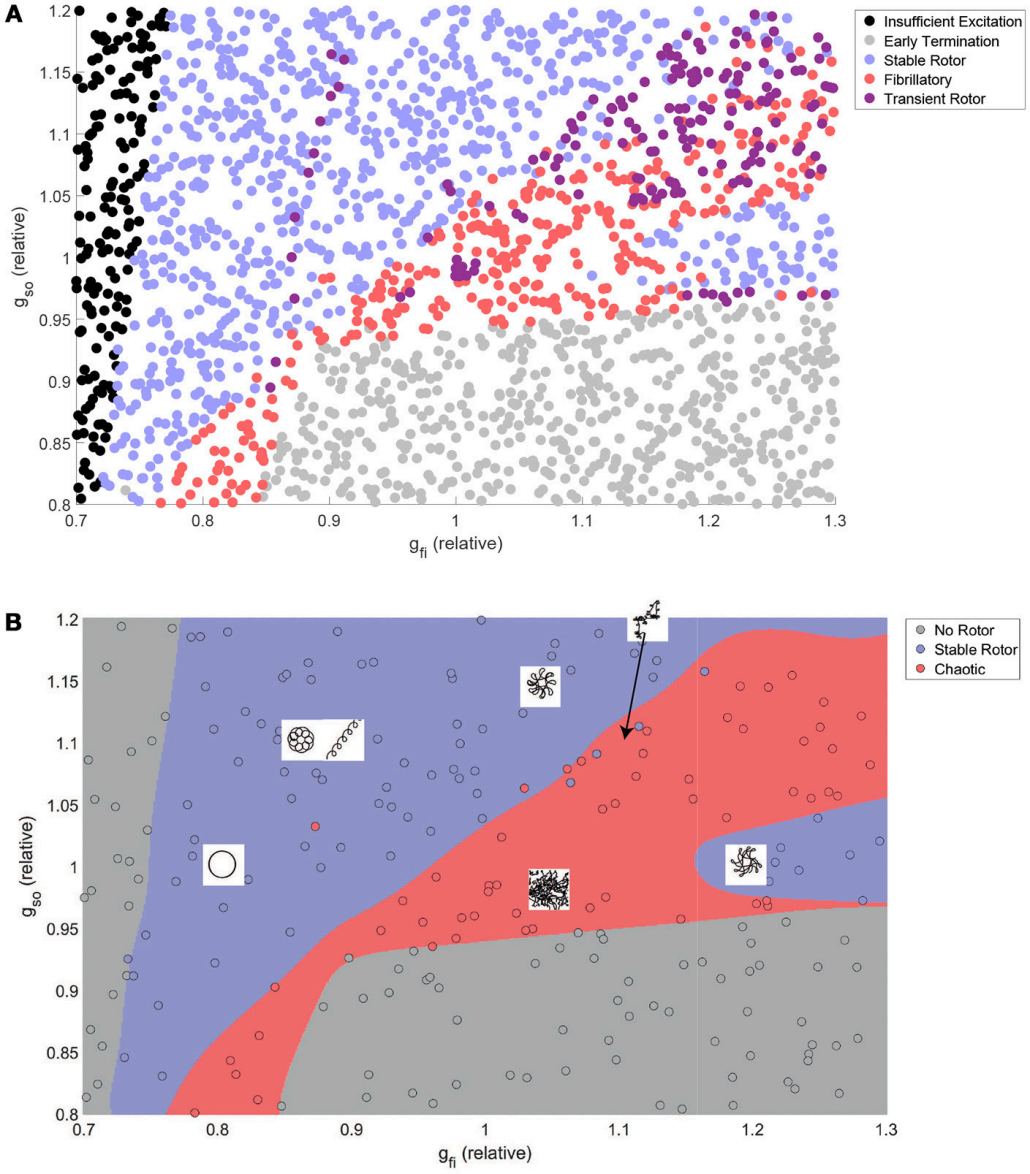
The class of re-entrant behavior exhibits complex dependence on ionic properties, but is well predicted by SVM classification. **(A)** Training and test data after classification using the rules in Table 2. Distinct parameter regimes that correspond to the different behaviors can be observed, but with small numbers of data points, predominantly in the fibrillatory region, that disrupt clean separations of the parameter space. **(B)** Combination of rotors that annihilate themselves with those that exhibit wave breakup into a single “chaotic” classification allows high-accuracy prediction of rotor behavior by an SVM classifier model. Also displayed are schematic diagrams indicating the different types of rotor path that can be generated in various regions throughout the parameter space.

Figure 3A shows how the automatically-detected class of rotor behavior changes in response to differences in the excitability of tissue (via *g*_*fi*_), and the rate of repolarization (via *g*_*so*_). Well-defined regions of the parameter space that correspond to the different behaviors can be clearly observed, but no boundaries can be drawn to separate the different classes in the top right of the parameter space. This issue is largely addressed by combining the “fibrillatory” and “transient rotor” classes together into a single “chaotic” rotor class, after which SVM classification successfully identifies the different regions and attains an accuracy of 96% on the data not used for training. The classifier model is thus appropriate for use in defining boundaries for partitioned emulation.

**Figure 4:**
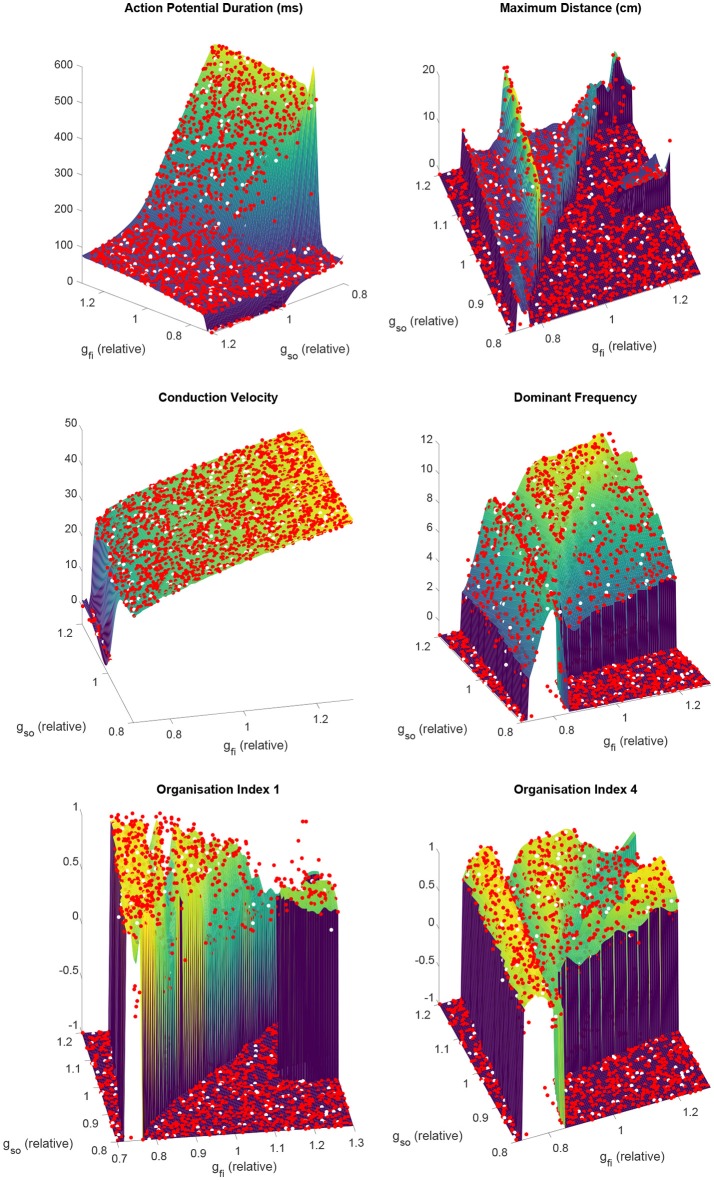
Partitioned emulation successfully captures the complex dependencies of important descriptors of re-entry on the electrophysiological substrate. The response surfaces predicted by the partitioned emulator for each biomarker are shown, along with the training data (red) used in their construction. Null values for biomarkers are denoted by a value of −1. Performance can be judged by considering the unseen test data (white), which demonstrate great agreement with the surfaces that indicate the emulator's predictions. This is achieved despite the complex and non-monotonic nature of the dependence of these biomarkers on the two varied parameters.

Despite very complex dependencies of the spatial biomarkers upon the values of these two channel densities, our partitioned emulation approach is able to very successfully capture the response surfaces implied by the data (Figure 4), allowing prediction of these important re-entrant properties at any point in the parameter space. The accuracy of these predictions is confirmed by comparing the data points not used in training against the emulated surface at those points, with the greatest majority of points falling very close to the line of equality (Figure S1). Importantly, the use of our partitioned emulation technique proves to significantly improve accuracy as compared to a traditional GP emulation approach using only a single emulator.

Training of the set of partitioned emulators required less than seven minutes, but the cost of generating the 2,000 simulations for use as training and test data required more than 2,000 h of computational time (albeit spread across multiple cores on our high performance computing platform). The time required for the emulator to generate predicted biomarker values at a set of 2,000 additional points randomly sampled across the parameter space was 0.23 s, indicating a speedup of eight orders of magnitude when considering serial implementation. Even making good use of supercomputer architecture, the speedup was about six orders of magnitude, indicating the power of emulation for studies of variability in cardiac electrophysiology models. These immense savings offered by the emulator allow further simulations to be generated almost immediately, enabling analyses such as the one that follows for a larger number of variable parameters.

Given the cost of generating the initial data used to construct our partitioned emulators, a natural question is how classifier and emulator performance depend upon the amount of training data used. In terms of accurately predicting the classes of the data reserved for testing, the strong performance of the SVM classifier remained even when using much less data for training (Figure S2). However the more data that is used, the better the precise locations of the boundaries between classes can be determined. Emulator performance depended strongly on the performance of the classifier, and 600 pieces of training data produced emulators almost as accurate as those trained with 1,800 pieces of data. It should be noted, however, that when a larger number of parameters is varied, a greater amount of training data will likely be required to successfully resolve classification boundaries and response surfaces.

#### 3.2.2. Capture of electrophysiological dynamics

The emulated surfaces for the biomarkers defining excitation wavelength, CV, and APD, demonstrate intuitive responses, to an extent, to variability in the two considered current densities (Figure 4). CV depends especially on the velocity of the AP upstroke, and so is especially sensitive to the conductance of the fast inward current (*g*_*fi*_) and only very slightly impacted by the conductance of the slow outward current (*g*_*so*_). APD depends strongly on the strength of the slow outward current, but importantly only above some critical level of fast inward current density. This effect arises because only sufficiently strong excitations are able to drive the AP upstroke beyond the activation threshold of the model's equivalent of the Ca^2+^ current. “Incomplete” excitations that fail to significantly activate this current result in very short APs, the duration of which are not strongly affected by either *g*_*fi*_ or *g*_*so*_.

As the conductance of the fast inward current is increased, a shift of rotor trajectories from circular to epicycloidal, then hypocycloidal paths is well established (Efimov et al., 1995; Fenton and Karma, 1998). We observe this same behavior (see example trajectories in Figures 1B, 3B), with such information encoded in the maximum distance and OI_1_ biomarkers. Specifically, the tightening of circular trajectories results in a decrease in core size until trajectories become epicycloidal, at which point sizes start increasing again, peaking for the case of traveling helices that collide with the boundary before paths become hypocycloidal. OI_1_ has a maximal value for the circular paths, decreased for epicycloidal and hypocycloidal trajectories, with severely reduced values for traveling helices and the high-excitability cases where trajectories meander much more.

The frequency of re-entry, controlled by the angular velocity of a spiral wave's core, depends on both the availability of excitable tissue and the speed at which re-entry can propagate into this tissue. Thus tachycardic severity increases in response to an increase in either of *g*_*fi*_ and *g*_*so*_. However, the nature of this increase depends on the type of rotor-driven re-entry, with circular rotor cores very sensitive to tissue excitability while rotors with hypocycloidal or meandering trajectories are barely affected. The final biomarker, OI_4_, successfully identifies what we term rotor “stability,” consistently taking on higher values when a rotor remains fixed in a single general location, as opposed to meandering or devolving into fibrillation. This biomarker thus serves as an indicator for the risk of wave breakup.

Several interesting observations can be made from the initial data presented up to this point. Figure 3 shows that rotor annihilation by antagonizing the outward current invariably involves first crossing through the chaotic regime. This suggests that if such treatments fail to sufficiently increase the critical length, and hence destroy a re-entry completely, they may instead trigger fibrillation, an effect we explore further in the analyses that follow. The sharp increase in maximum distance and decrease in OI_1_ at the boundary between the stable and chaotic regions also suggests that rotor meander in general is an indicator for increased susceptibility to wave breakup. Lastly, we observe that for all biomarkers (less marked perhaps for CV) the dependence on either of the two parameters depends strongly upon the value chosen for the other. Thus we must consider variability in all properties of interest at once, in order to ensure that the conclusions drawn are not significantly biased by the specific set of base parameter values chosen. This is one of the advantages we achieve in the following section via emulation.

### 3.3. Classification and emulation identify key ionic effects underlying functional re-entry

#### 3.3.1. Partitioned emulation is an important tool for understanding variability in cardiac electrophysiology models

The previous section demonstrated the importance of allowing different electrophysiological properties to vary simultaneously, in order to properly understand how the effects of variation in these different properties (including the effects of drug treatments) together determine re-entrant behavior. However, when our full set of electrophysiological properties are all allowed to vary, we lose the ability to use forward simulation to properly explore the parameter space due to its high dimensionality. Here the SVM classifier model and associated emulators become invaluable tools, to enrich the data and to make the calculation of the main effects of each parameter possible, thus allowing us to quantify how each individual ionic property contributes to the type of arrhythmia that develops, as well as its relative severity. We select the majority of the *in silico* data (3,200 out of 3,500 points) to be used for training the classifier model and emulator, in order to attain the best performance possible when the emulator is then used outside of the dataset to calculate the main effects of the parameters.

Although the predictions of the emulators and classifier model they depend upon cannot be simply visualized when these many parameters are varied, the test data not involved in the training process can be used to evaluate their performance. The increased dimensionality makes both the classification and emulation problems significantly more difficult, but the SVM classifier still achieves an accuracy of 81%. Emulation performance is also diminished as compared to the lower-dimensional problem, but remains good enough that the emulator is suitable for the efficient and automatic extraction of hidden data trends (Figure S3). Use of our partitioning technique resulted in more than a fivefold decrease in root mean square error, as compared to traditional GP emulation. Importantly, the differences incurred by emulation are not strongly biased, with no consistent under-estimation or over-estimation. The strong performance of the classifier and emulator on the two-parameter problem suggests that the reduction in performance for the eight parameter problem is simply due to the increased amount of training data required to fully resolve such complex dynamics across a high-dimensional space, and thus may be improved simply by further additional runs of the simulator.

#### 3.3.2. Slower recovery of fast inward channels can increase arrhythmic risk

Using our emulators to rapidly evaluate Equation (3), we quantify using mean effects how the different parameters controlling excitability and its recovery impact upon the important features of our simulated re-entries. We re-iterate that these insights are not the same as would be obtained by simply varying each individual parameter in turn, but instead represent the overall effects of the parameters upon variability in the others. This improves the generalizability of the conclusions we draw in the face of both physiological variability and uncertainty in the most appropriate base values of the parameters in a model.

**Figure 5:**
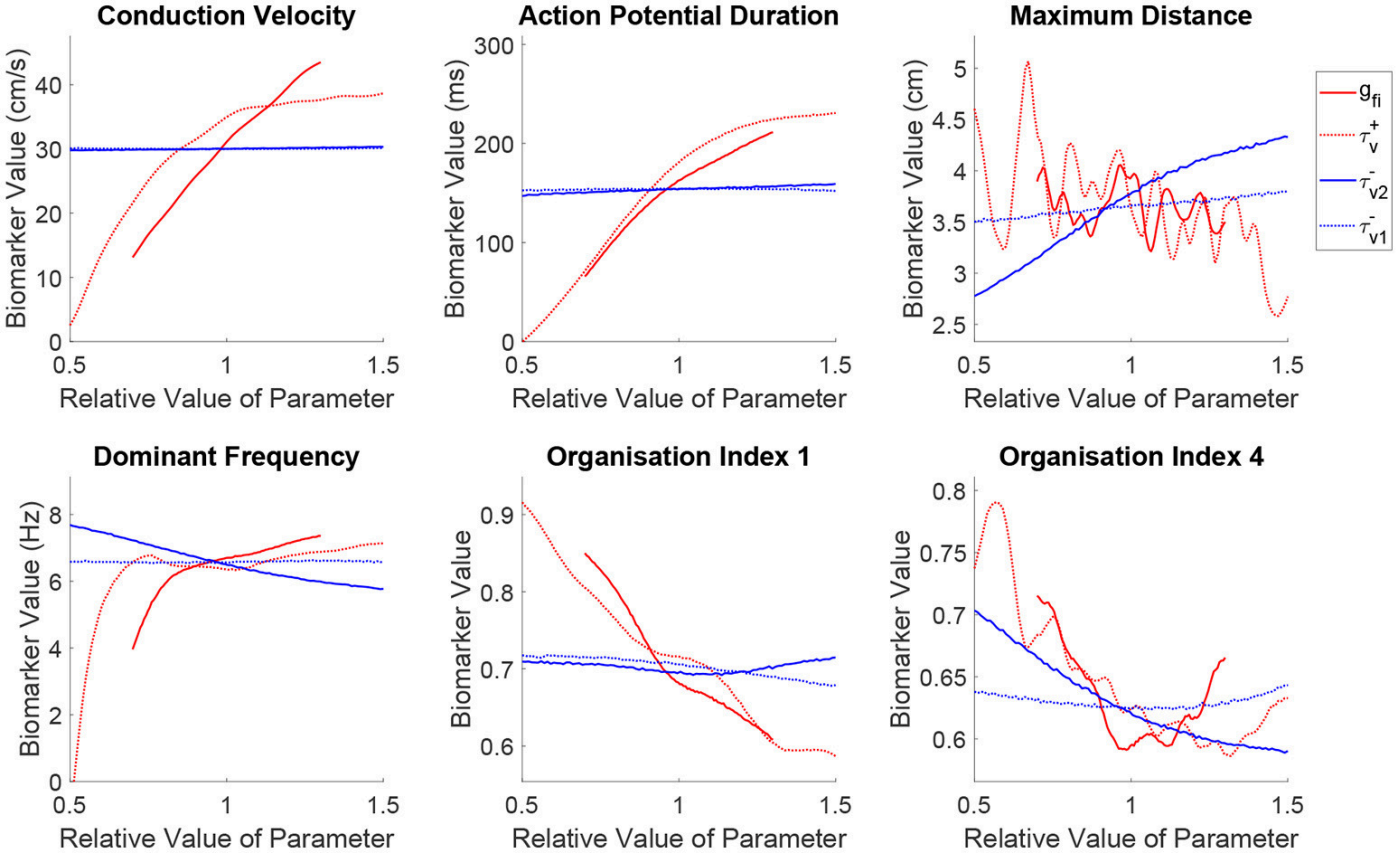
Sustained re-entries respond more consistently to the recovery of tissue excitability than to fast inward current amplitude, but slowed recovery incurs an increased risk of wave breakup. Main effects of the parameters controlling tissue excitability and its recovery. Excitability strongly controls the shape of wavetip trajectories (OI_1_) and rotor stability (OI_4_), but has less consistent effect on the critical amount of tissue for sustained re-entry (maximum distance) and re-entrant frequency. On the other hand, by modulating ERP, recovery of fast inward channels does predictably control core size and frequency in sustained re-entries, but notably, slower recovery (increased ERP) is linked to an *increased* risk of wave breakup.

Figure 5 shows how the spatial biomarkers we use to quantify re-entry properties depend on the parameters controlling tissue excitability. Targeting the current density of the fast inward channels (*g*_*fi*_) largely impacts CV by modulating upstroke velocity and AP amplitude, as well as regulating APD owing to the effect discussed previously where only sufficiently strong excitations are able to fully activate the slow inward current (Fenton and Karma, 1998). An increased fast inward channel density therefore increases the excitation wavelength by simultaneously augmenting APD and CV. However, the dependence of the maximum distance traveled by a rotor on this property is quite complex, where the multiple peaks in this biomarker correspond to shifts through different types of rotor trajectories (circles into epicycloids into helices into hypocycloids, as also observed in the two-parameter case), here further complicated by the variability in other cell properties. Increased channel density also tends to increase the complexity of the rotor trajectory (lower OI_1_), as the faster propagation causes the rotor to attempt to rotate more quickly (increased DF), promoting tip-wake interactions. The risk of breakup (lower OI_4_) is however reduced, as in these situations the rotor tip makes longer linear runs along lines of conduction block. Note, however, that both the complexity of re-entry and the risk of breakup are reduced (higher OI_1_ and OI_4_, respectively) for decreasing values of *g*_*fi*_, in spite of this implying a reduction of the excitation wavelength. This corresponds newly to situations of weak excitability, where the slow inward current can never activate and the APD rate-dependence is significantly lost, preventing repolarization heterogeneities that can lead to wave breakup.

The discussion above additionally applies to fast inward current inactivation ($\tau_{v}^{+}$), given its concomitant role in modulating upstroke velocity and AP amplitude. However, a slower inactivation of the fast inward current (larger $\tau_{v}^{+}$) results in a more marked decrease of the maximum distance traveled by a rotor, in spite of yielding an equivalent increase of the excitation wavelength and DF. This increases the chances of wavefront-waveback interactions, further increasing the complexity of the rotor trajectories (lower OI_1_), and risk of breakup (lower OI_4_).

Increasing the ERP by slowing the recovery of the fast inward channels (larger $\tau_{v2}^{-}$) makes it harder for rotors to rotate, with re-entrant paths occupying more space and triggering the tissue at a slower rate in this case. This is captured by the mean effects of $\tau_{v2}^{-}$ for maximum distance and dominant frequency, and is achieved in the absence of any changes on either steady-state CV or APD. However, the organization indices show less expected responses to variability in this parameter. The complexity of rotor trajectories (OI_1_) show no significant dependence on recovery of excitability. More remarkably, the risk of breakup (lower OI_4_) actually *increases* in this case, as the slower recovery of excitability lengthens the timing window in which the fast inward channels are only partially recovered and weak excitations can be triggered. These weak excitations fail to completely activate the slow inward current, creating large APD discrepancies across the tissue and thus an increased repolarization heterogeneity and hence fibrillation. Importantly, this result challenges the hypothesis that increased ERP may be protective in all cases against fibrillation by increasing the excitation wavelength (Smeets et al., 1986; Lee et al., 2013).

#### 3.3.3. APD modulation of cardiac wavelength directly controls key re-entry properties, but not risk of breakup

**Figure 6:**
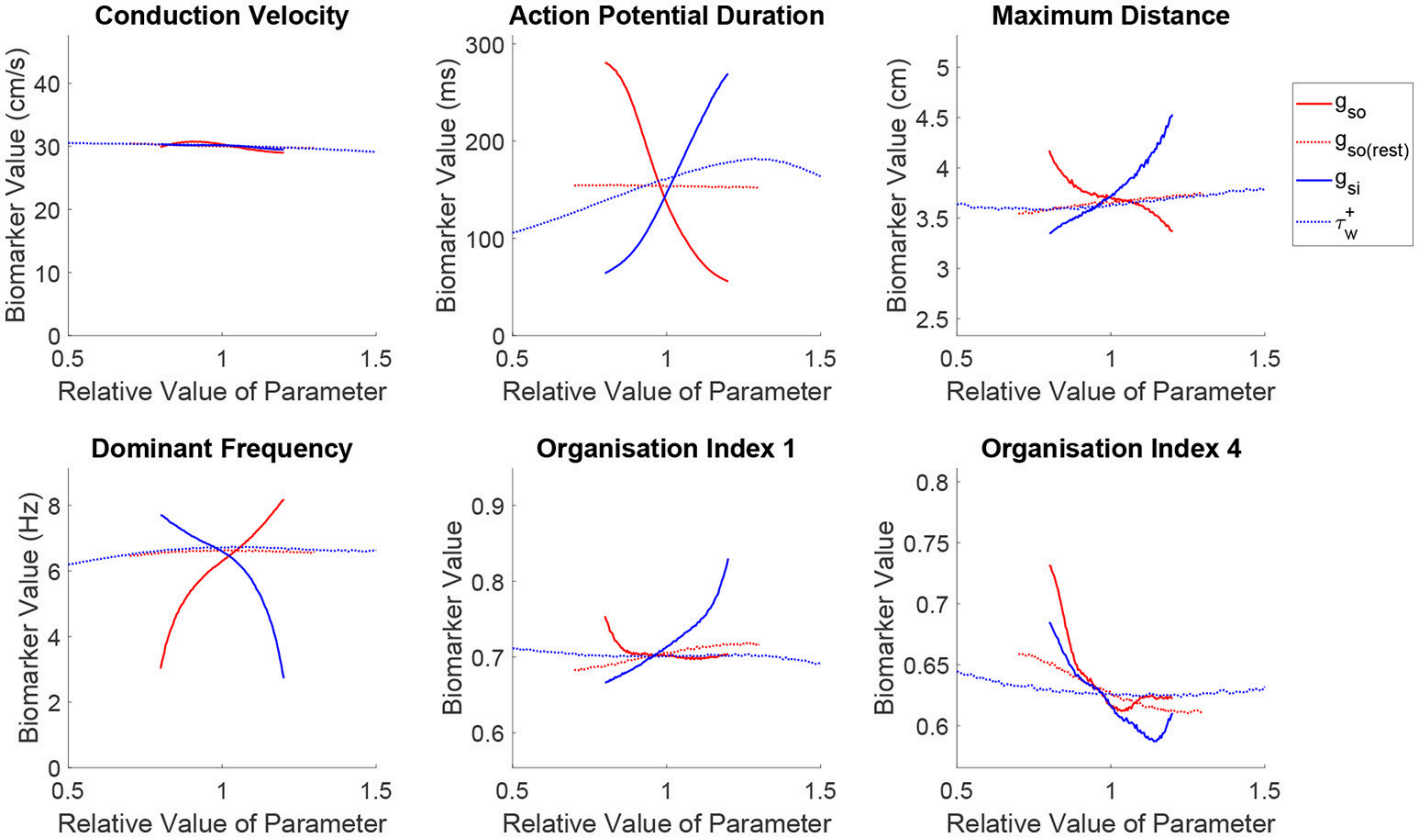
Slow inward current exhibits important effects on re-entrant behavior beyond its control of APD. Main effects of the parameters controlling tissue repolarization. The conductances of the two currents that control APD are seen to strongly affect the critical size and frequency of rotor-driven re-entry, according to their modulation of ERP. However, the strengths of both of these currents exhibit a similar effect on rotor stability despite their opposing influences on APD/ERP, highlighting the importance of subtler restitution effects controlling the risk of breakup. The slow inward current also emerges as an important controller of the complexity of wavetip trajectory in the case of stable spiral waves.

Just as in the previous section, we use the mean effects to quantitatively explore the impacts of variability in the parameters controlling repolarization on our set of spatial biomarkers, with the results visualized in Figure 6. Variability in any of the cell properties governing repolarization has essentially no effect on CV, whilst APD is strongly affected by the conductances of the two primary currents active during repolarization (*g*_*so*_ and *g*_*si*_). Notably, the inactivation of the slow inward current ($\tau_{w}^{+}$) has a not insignificant effect on APD (slower inactivation of the slow inward current prolongs the AP), but this parameter proves less important in controlling any of the considered re-entry biomarkers.

Variability in the primary repolarization current modulates APD and by extension ERP, determining the availability of excitable tissue for a spiral wave's tip as it rotates. A weaker repolarization (smaller *g*_*so*_) increases APD, resulting in longer critical lengths and slower re-entrant DF. Variability in repolarization exerts stronger control and with more monotonic trends over these biomarkers as compared with excitability. ERP then modulates the overall size and angular velocity of these trajectories, which for reduced repolarization (smaller *g*_*so*_) translates into a smaller complexity of rotor trajectories (increased OI_1_) and reduced risk of fibrillatory and other chaotic behaviors (increased OI_4_), nicely fulfilling the excitation wavelength hypothesis.

The effects described above are largely mirrored by the slow inward current, corresponding to its role in opposing repolarization. An increased current density (larger *g*_*si*_) prolongs the AP plateau and therefore APD/ERP, increasing the critical size and decreasing the DF of re-entry and exerting a more dominant effect on the shape of rotor trajectories (increased OI_1_) compared to the repolarization currents. However, when the conductance of the slow outward current is increased, a sharp decrease in rotor stability is observed (reduced OI_4_), followed by an approximately level trend, which contradicts the excitation wavelength hypothesis given the increased ERP. The de-stabilizing effect of increased conductance of the slow inward current can be explained by the increased rate-dependence of the tissue, exacerbating spatial dispersion of repolarization that may arise and thus promoting wave breakup. In the context of rotor stability, this control over rate dependence is seen here to be more important than the current's control over APD, explaining some of the failure in using APD and CV to predict the class of re-entrant behavior that has been previously discussed.

#### 3.3.4. Critical windows of excitability determine risk of breakup

Our main effect analysis implicates the AP upstroke and the strength of the slow inward current as key factors controlling the likelihood that a rotor-driven re-entry devolves into fibrillation, along with an increased risk associated with slow recovery of excitability. Using the classifier model, we now explore precisely how these cell properties together determine when such chaotic dynamics arise. Visualizations are presented in the form of two-dimensional maps (non-specified parameters held to base values), although our classifier automatically takes the simultaneous variability in all parameters into account.

**Figure 7:**
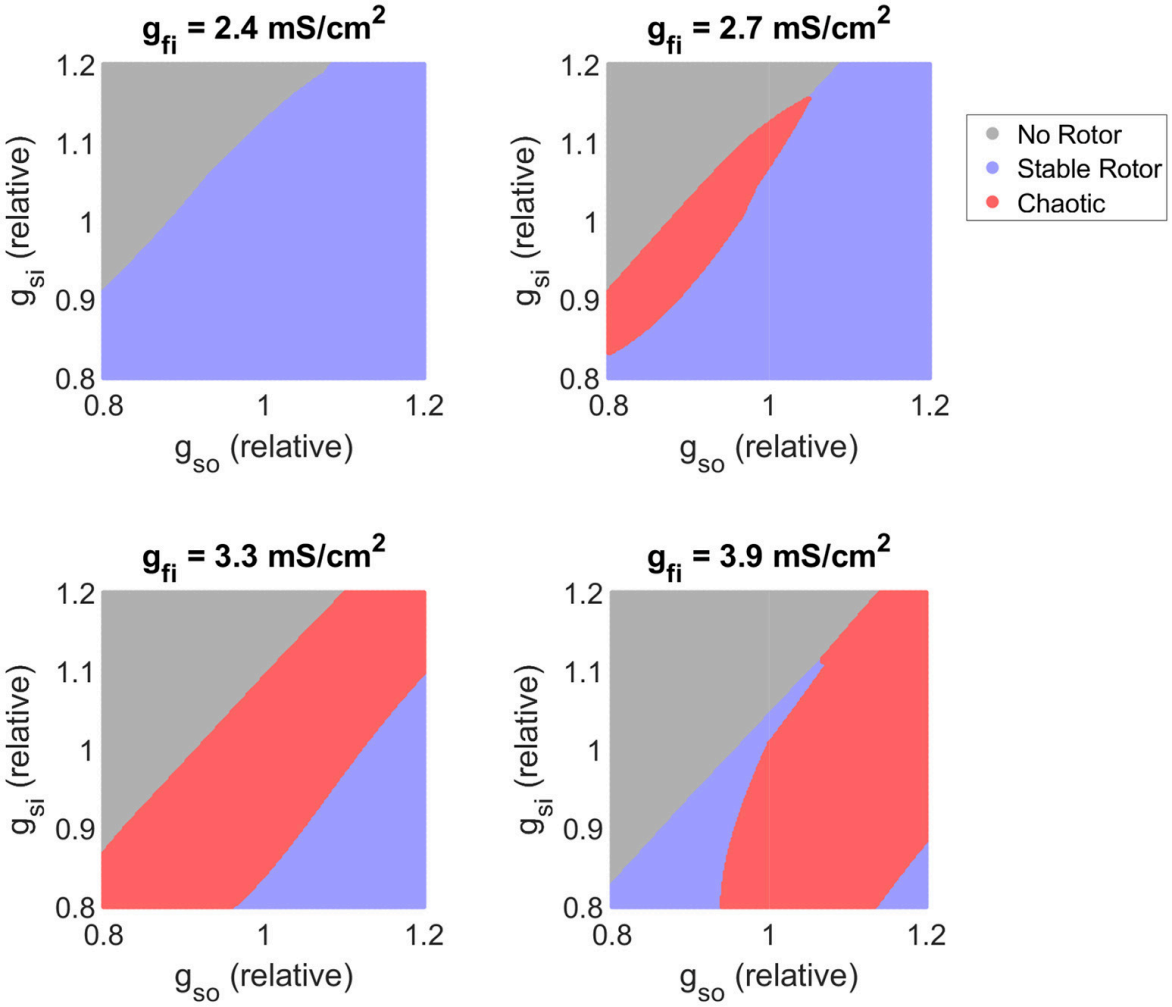
Variability in excitability critically determines the balance of slow current conductances that produces chaotic re-entrant behaviors. Parameter maps as predicted by the classifier model. As tissue excitability (as controlled by the conductance of the fast inward current) increases, the balance of inward and outward currents that triggers chaotic re-entry behaviors shifts, highlighting its importance in interpreting APD in terms of fibrillation risk. Increased slow inward current is here consistently associated with an increased risk of wave breakup.

Figure 7 shows how changes in excitability, as modulated by the conductance of the fast inward current, affects the balance of slow inward and outward currents in determining rotor stability. As implied by our former analysis of the main effects of *g*_*fi*_, when excitation is too weak, all formed rotors remain stable as the slow inward current is not fully activated, diminishing the rate-dependence of the tissue. As the density of the fast inward current increases, the region of chaotic re-entrant behaviors determined by the balance between the slow inward and outward currents (which together primarily determine APD except when excitability is too weak) shifts significantly. This confirms that APD/ERP does not serve as a suitable predictor for the risk of fibrillation, and given the complex dependency on excitability we see here and in the main effects (Figure 5), neither does the cardiac wavelength as the product of CV and ERP. On the other hand, regardless of the strength of fast inward current, increased slow inward current is seen here to always carry a greater risk of wave breakup (provided APD is not so long that a rotor fails to be induced). This points to the importance of Ca^2+^ antagonism as a potential anti-arrhythmic mechanism (Merillat et al., 1990).

**Figure 8:**
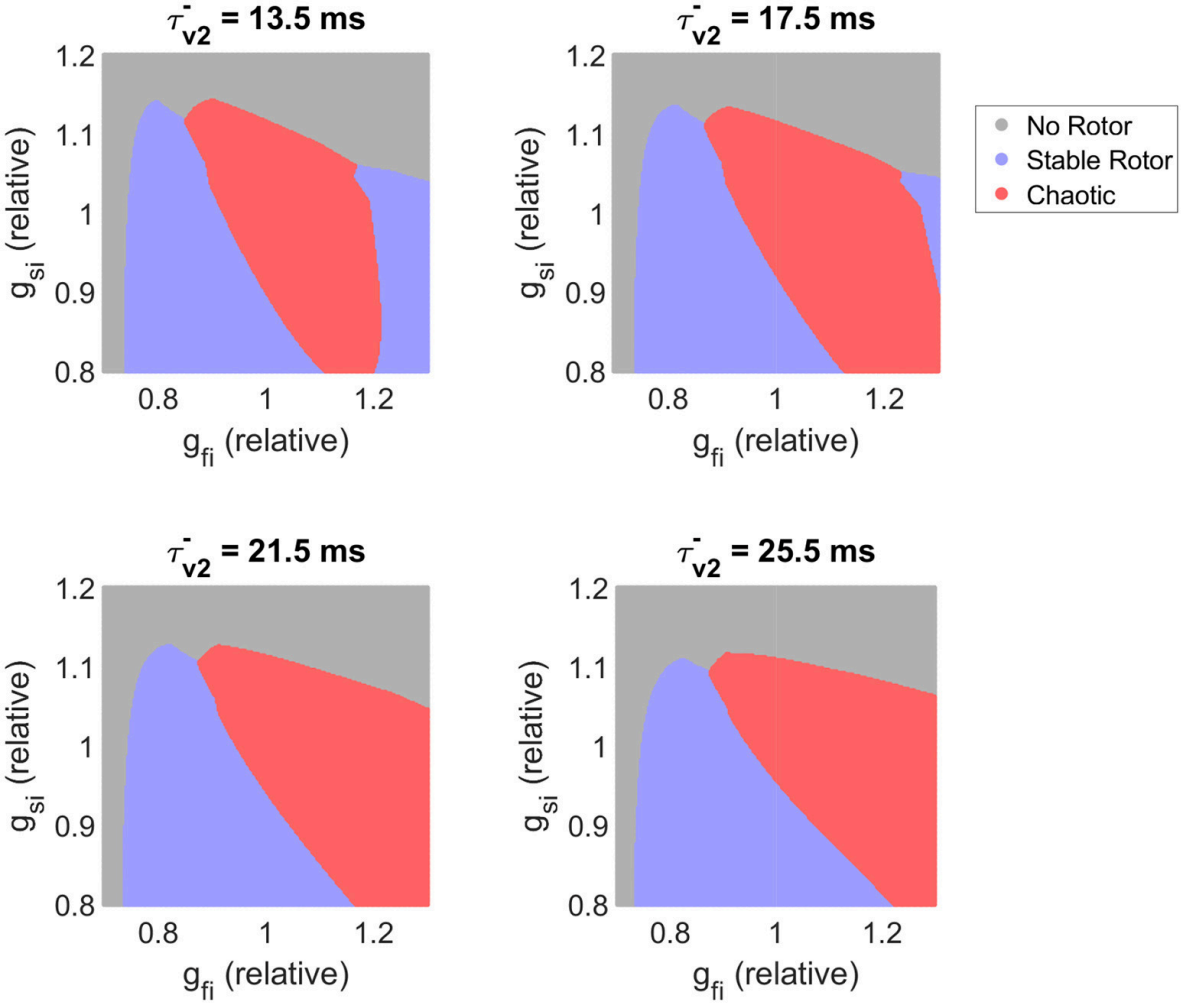
Slower recovery of excitability widens the critical window of fast inward current density that can trigger breakup, and shifts this window to higher amounts of current density. Parameter maps as predicted by the classifier model. For the lower values of $\tau_{v2}^{-}$ plotted, the existence of a critical window of fast inward current strength associated with highest risk of wave breakup is clearly seen. As slow inward current increases in strength, this window widens and shifts to lower *g*_*fi*_ values. Slower recovery of fast inward channel availability (larger $\tau_{v2}^{-}$) corresponds on the whole to destabilization of functional re-entry, but the specific effect depends also on the conductance of this current.

We next further explore our observation that slower recovery of excitability corresponds to decreased re-entrant stability, despite increasing ERP. Figure 8 visualizes the effects of different time constants of fast inward channel recovery ($\tau_{v2}^{-}$) on how the stability of re-entry is modulated by the two factors previously identified as critical determinants of fibrillatory behavior (conductances of the fast inward and slow inward currents). These parameter maps further support the results of the previous section, namely that increased slow inward current increases risk of fibrillation and that there exists a critical window of fast inward current density for initiation of wave breakup. The effect of delaying the recovery of excitability is clearly seen in shifting the high end of this critical window to higher values of *g*_*fi*_. The stable re-entries that are affected by this are rotors with cores making long runs followed by rapid rotations (Figure 3), characteristic of human ventricular rotor dynamics (Bueno-Orovio et al., 2008). Finally, we note that when fast inward current inactivation ($\tau_{v}^{+}$) is varied instead of *g*_*fi*_ (as the additional main determinant of tissue excitability), the resulting classification maps display all of the same key behaviors (Figure S4). This strongly suggests that the existence of critical windows of excitability is not only limited to the density of the fast inward current (as corroborated by the mean effect analysis of CV and OI_4_ presented in Figure 5), with delayed fast inward inactivation as an additional pro-arrhythmic mechanism of risk of wave breakup.

## 4. Discussion

In this work, we provide new mechanistic insights on the important interplay between cardiac refractoriness and arrhythmic risk, by identifying that both a slower recovery of fast inward channels or an increase of slow outward currents can promote chaotic rotor dynamics and wave breakup. This is in spite of increased ERP in each case, with arrhythmic risk mediated by an enlarged dispersion of repolarization associated with rate dependence. Our insights were only made possible by the development of a novel approach to constructing partitioned emulators synergistically coupled to cardiac electrophysiological simulations. This allowed not only data enrichment, but the automatic detection, classification, and analysis of different re-entrant behaviors, and quantitative determination of how the different electrophysiological properties modulating tissue excitability and refractoriness control the behavior of rotor-driven arrhythmias.

Steady-state properties of the tissue, APD, and CV, emerged as good predictors only for the critical amount of tissue required to sustain a rotor upon induction, but unable to identify chaotic regimes or susceptibility to wave breakup. The slope of the APD restitution curve, which quantifies the adaptation of APD to the pacing rate, has been well studied as a potential means of predicting rotor break-up (Nolasco and Dahlen, 1968; Karma, 1994; Qu et al., 2000; Nash et al., 2006), appealing because it can also be readily measured experimentally. However, flattening of this slope has been proposed as both an anti-arrhythmic target (Qu et al., 1999; Garfinkel et al., 2000) and a potentially pro-arrhythmic property (Franz, 2003), and a steep slope provides no guarantee of fibrillatory activity (Cherry and Fenton, 2004). Furthermore, recent studies have also demonstrated that APD restitution slope is primarily determined by steady-state APD (Bányász et al., 2009; Bárándi et al., 2010), and that normalizing the restitution curve as percentual changes of steady-state APD abolishes the differences in restitution slope in a variety of interventions (Shattock et al., 2017). This reinforces the role of steady state APD (and therefore excitation wavelength) for mechanistic investigation of re-entry, but also highlights the importance of considering the impacts of variation in cell-level properties directly as we do here, instead of just in terms of their modulation of tissue-level properties.

Variability in fast inward channel conductance and inactivation had an inconsistent effect on the critical size of rotors and for the most part little effect on the dominant frequency, despite directly controlling CV. These properties proved the primary determinant of wavetip trajectory for stable rotors, and their inconsistent effect on core size is thus explained by the shifting through the several different types of trajectory. Only in the specific case of re-entries driven by circular rotor cores could rate control be achieved by slowed conduction. On the other hand, core size and frequency of re-entry depended much more consistently on the availability of excitable tissue (ERP), a function of both the recovery of excitability and the rate of repolarization. These results agree with the relative success of anti-arrhythmics that prolong ERP in treating ventricular tachycardias (Haverkamp et al., 1997; deSouza et al., 2015), the sustainability and severity of which depend critically on these two biomarkers. However, this effect must be considered in tandem with the potential pro-arrhythmic effects of such treatments that we discuss subsequently.

Depending on the electrophysiological properties of the simulated tissue, we observed wave breakup into fibrillation (potentially transitory or sustained for the full duration of simulation). Increased slow inward current was consistently associated with wave breakup, but increased slow outward current failed to show the opposite effect. This strongly suggests that it is not physiological variability in APD/ERP that controls breakup, and instead implicates the importance of slow inward currents in defining the extent of rate-dependence in the tissue and hence promoting dispersion of repolarization. In fact, we do note that some treatments that decrease ERP by antagonizing slow inward currents can trigger a reversion from ventricular fibrillation by reducing spatial dispersion of repolarization (Kimura et al., 2005; Bossu et al., 2018), consistent with these conclusions. Additionally, we observed a critical window of tissue excitability as a function of fast inward channel density that presents the highest risk of wave breakup. When excitability is too low, complete activation of the slow inward current does not occur regardless of activation timing, preventing spatial dispersion of recovery and hence breakup. When excitability is too high, the rotor tip exhibits longer linear runs between its rotations, and thus has reduced opportunity to interact with its own wake, as well as a much-increased probability of colliding with tissue boundaries and hence annihilating itself before an episode of fibrillation can occur.

Reduced sodium channel availability by slow recovery of the fast inward channels emerged as a significant promoter of chaotic behavior like wave breakup, by increasing the aforementioned dispersion of repolarization associated to differential activation of slow inward currents. Such a mechanism is indeed expected to be of greater importance in the ventricles, where Ca^2+^ currents play a greater role in modulating rate dependence as compared to the more triangular APs in the atria (Sánchez et al., 2012). These results thus mechanistically explain the good performance of class Ic anti-arrhythmics in terminating atrial fibrillation, but at the cost of increased susceptibility to ventricular arrhythmias. This is for example the case of pilsicainide, a class Ic agent with slow recovery kinetics, successful in the clinical management of atrial fibrillation (Kanki et al., 1998; Fukuda et al., 2011) in spite of reports of its involvement in precipitating ventricular Torsade de Pointes (TdP) arrhythmias and sudden cardiac death (Nakatani et al., 2014), as further corroborated by its “possible risk” TdP category in the CredibleMeds database (Woosley and Romer, 1999). These results also hold for the controversial role of flecainide, another class Ic agent especially successful in the treatment of atrial fibrillation (Wang et al., 1992; Aliot et al., 2011), but with “known risk” TdP category (Woosley and Romer, 1999; Nasser et al., 2015), although its potent inhibition of potassium repolarizing currents can also mediate its pro-arrhythmic profile (Paul et al., 2002; Melgari et al., 2015; Passini et al., 2017).

Our findings with regard to the stability of functional re-entries were further supported and refined by the predictions of our SVM classifier model. After identifying the important ionic properties underlying risk of wave breakup, visualization of the classifier's predictions confirmed the importance of slow inward current density, the existence of critical windows of excitability, and the inability to rely upon APD or excitation wavelength as biomarkers for the risk of wave breakup. Furthermore, the pro-arrhythmic potential of slowed recovery of excitability was suggested to be of stronger importance for rotor cores that move via a pattern of a long run followed by a tight rotation, potentially differentiating the cases discussed above where class Ic anti-arrhythmics may or may not induce fibrillation in the ventricles.

Importantly, each of these observations on properties of re-entry and susceptibility to breakup have been obtained while considering variability across a large number of electrophysiological properties. The emergence of these behaviors across significant variation in the parameters suggests that they are in fact core electrophysiological behaviors, and not simply limited to the regime implied by the base parameter values considered in the FK model. Promisingly, a recent study using a biophysically-detailed model to explore variability in ionic current conductances with regard to the meander of rotor-driven re-entries in the atria also identified the Ca^2+^ and Na^+^ current densities as the most important parameters (Liberos et al., 2016), agreeing with their observed importance here, and reinforcing the potential of reduced ionic models to capture tissue-level dynamics of cardiac electrophysiology.

Methodologically, our work is probably the most comprehensive computational investigation to date of the complex interplay between cardiac excitability and refractoriness in modulating rotor-driven arrhythmic behavior and susceptibility to breakup, comprising more than 5,000 forward simulations of long-lasting arrhythmic episodes, further enriched to 80 million electrophysiological scenarios by the innovative application of emulation to this field. Despite the existence of clear bifurcations and chaotic regimes, our classification and emulation approaches proved capable of predicting with good accuracy the nature of rotor behavior and important spatial biomarkers characterizing rotor-driven re-entry, even with a relatively large number of electrophysiological properties allowed to vary. The highly complex and non-monotonic response surfaces for spatial biomarkers of re-entry presented in this study further illustrate that the dynamics of arrhythmia and fibrillation in cardiac tissue cannot be predicted by simple fits to the data such as linear regression. On the other hand, these are accurately and automatically captured by the use of emulation, also serving to eliminate representation bias in data analysis and interpretation. Altogether, our work exemplifies a synergistic combination of supercomputing, machine learning, and advanced statistical methods, pushing the frontiers of big data applications for investigations on cardiac electrophysiology.

Due to our specific focus on rotor dynamics, we have used here the reduced FK model (Fenton and Karma, 1998), which accurately captures the restitution properties of cardiac tissue but does not contain biophysical representations of each of the many currents that govern the APs of cardiac cells. Now that our approach to emulating cardiac electrophysiology models has been validated, further exploration of the impacts of variability in tissue excitability and refractoriness could be obtained by applying these techniques to biophysically detailed models with full characterization of ion channel kinetics, which may offer additional insights into how different anti-arrhythmic agents could be expected to perform in a variable population. However, and in spite of its reduced complexity, it is important to note that predictions on mechanisms of wave instability using the FK model have been confirmed with more sophisticated models (Rappel, 2001; ten Tusscher and Panfilov, 2006). Another natural extension is the emulation of spatial biomarkers for simulations on anatomically accurate geometries, thus incorporating structural effects and making the cases where a rotor fails to develop due to collision with the domain boundaries much more physiologically relevant. Such extensions to the cardiac model would not require any adjustment to our method for emulation, beyond perhaps the creation of additional classifications to categorize any new patterns of model behavior that might arise.

In conclusion, we have demonstrated how emulation can be adapted to models that govern the complex spatiotemporal dynamics of re-entry in cardiac electrophysiology. We have used the great reduction in computational cost offered by emulation in order to further explore how variability in tissue excitability, repolarization, and post-repolarization refractoriness all affect whether rotor-driven re-entries are electrophysiologically supported, the likelihood that they exhibit wave breakup and the severity of the arrhythmias that they induce. This variability analysis did not require the fixing of key model parameters to specific values, making the results much more generalizable. This type of approach is of especial relevance in cardiac electrophysiology, where parameter variability is known to be important, and has a significant effect on the interpretation of both experimental and modeling studies.

## Author contributions

All authors contributed to the development of the presented methodology. BL, CD, and AB-O implemented the methodology and performed simulations. BL, KB, PB, and AB-O performed analysis of the results. All authors contributed to the drafting and refinement of the manuscript.

### Conflict of interest statement

The authors declare that the research was conducted in the absence of any commercial or financial relationships that could be construed as a potential conflict of interest. The reviewer RC and handling editor declared their shared affiliation at the time of the review.
