# Supplementary material for: Slow Recovery of Excitability Increases Ventricular Fibrillation Risk as Identified by Emulation
Source: Front Physiol. 2018 Aug 28;9:1114. doi: 10.3389/fphys.2018.01114 (PMC6121112; doi:10.3389/fphys.2018.01114)
Supplement: Supplementary file 1 [file Data_Sheet_1.PDF]

frontiers

Slow Recovery of Excitability Increases Ventricular Fibrillation Risk as Identified by Emulation

Brodie A. J. Lawson [1,*], **Kevin Burrage** [1,2], **Pamela Burrage** [1], **Christopher C. Drovandi**[1] **and Alfonso Bueno-Orovio** [2]

[1] *ARC Centre of Excellence for Mathematical and Statistical Frontiers, School of Mathematical Sciences, Queensland University of Technology, Brisbane, QLD, Australia*

[2] *Department of Computer Science, University of Oxford, Oxford, United Kingdom*

Correspondence*:
Brodie Lawson
b.lawson@qut.edu.au

SUPPLEMENTAL DATA

Full Ionic Model

The Fenton–Karma model represents the activation and repolarisation of cardiac cells as the net effect of three currents, each representing in a sense the movement of one type of ion across the cell membrane. The fast inward current, J_{fi} is responsible for the sharp upstroke that triggers the AP in response to an external stimulus, thus approximating the role of ion flow through fast inward Na^{+} channels in more biophysically detailed models. The slow outward current, J_{so}, is the model's only outward current and hence solely responsible for repolarisation, approximating the role of the combined effect of many K^{+} currents. Last is the slow inward current, J_{si}, which approximates the role of the plateau current carried by Ca^{2+} and acts to prolong the AP in a rate-dependent manner, recovering important restitution properties. The combined effect of the three currents is the total contribution of ionic effects to change in the membrane potential,

$$I_{\text{ion}} = -\left(J_{fi} + J_{so} + J_{si}\right),$$

with the three currents defined as

$$\begin{aligned}
J_{fi} &= -g_{fi}\, v\,(1-u)(u-u_c)H(u-u_c) \\
J_{so} &= g_{so(\text{rest})}\, u\, H(u_c-u) + g_{so}\, H(u-u_c) \\
J_{si} &= -g_{si}\, w\, \frac{(1+\tanh[k(u-u_{si})])}{2}.
\end{aligned}$$

Here $H(u)$ is the Heaviside step function, and is used to capture the voltage-dependent switching behaviours of the different currents without introducing additional variables or mathematical expressions that are more costly to evaluate. Two dependent variables v and w summarise the gating behaviour of the fast inward and

slow inward currents, respectively, and update dynamically according to the ordinary differential equations

$$\frac{dv}{dt} = \frac{(1-v)}{\tau_v^-}H(u_c - u) - \frac{v}{\tau_v^+}H(u - u_c)$$
$$\frac{dw}{dt} = \frac{(1-w)}{\tau_w^-}H(u_c - u) - \frac{w}{\tau_w^+}H(u - u_c).$$

Simply, both gating variables close at an exponential rate when the cell is active ($u \geq u_c$), and open at a different exponential rate when the cell is at rest ($u < u_c$). Furthermore, in order to better capture restitution properties for the conduction velocity, the time constant for v is further split up, as follows

$$\tau_v^- = \tau_{v1}^- H(u - u_v) + \tau_{v2}^- H(u_v - u).$$

Base parameter values and the ranges of variability are provided in the main document.

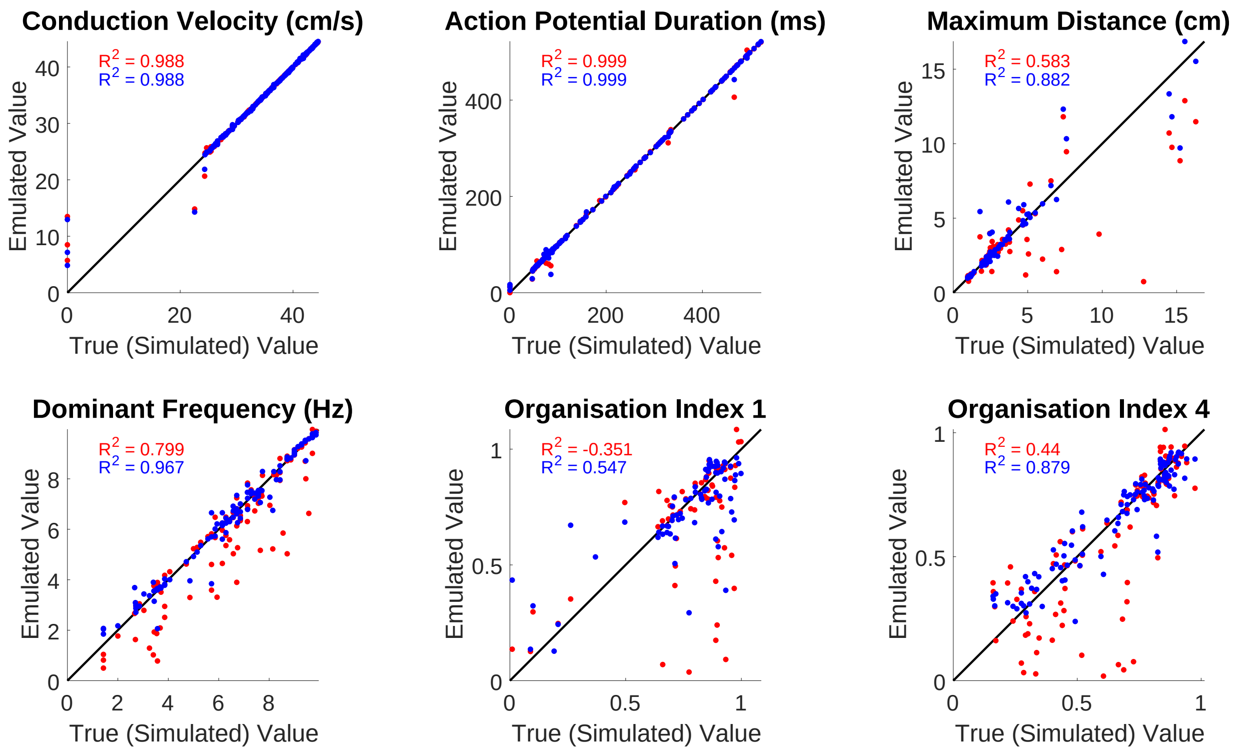

Figure S1. The performance of a partitioned (blue) and single (red) emulator on the two parameter problem, expressed by comparison between the unseen test data generated by the simulator and the emulator's predictions for those same locations in the parameter space. Only non-null and correctly classified values are displayed and used in R^2 value calculation. Partitioning the parameter space significantly improves emulator performance, with R^2 values better in all cases. The basic properties of excitation waves (CV and APD) are emulated exceedingly well.

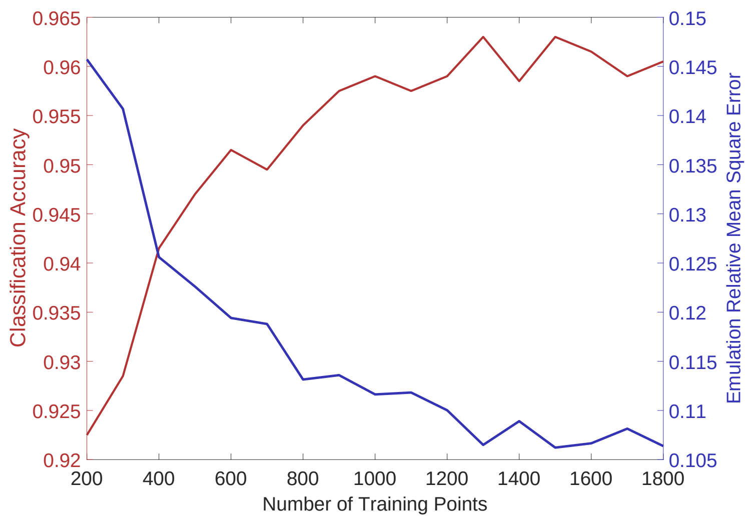

Figure S2. Strong performance is obtained even when smaller fractions of the *in silico* data are used for classifier and emulator training, but emulation accuracy is further improved using increased amounts of training data. Here relative root mean square error refers to the mean square error, scaled for each biomarker by the range of values for that biomarker, and then averaged over all biomarkers. Independent of the number of training points used, all performance measures were calculated using the same set of test points for consistency.

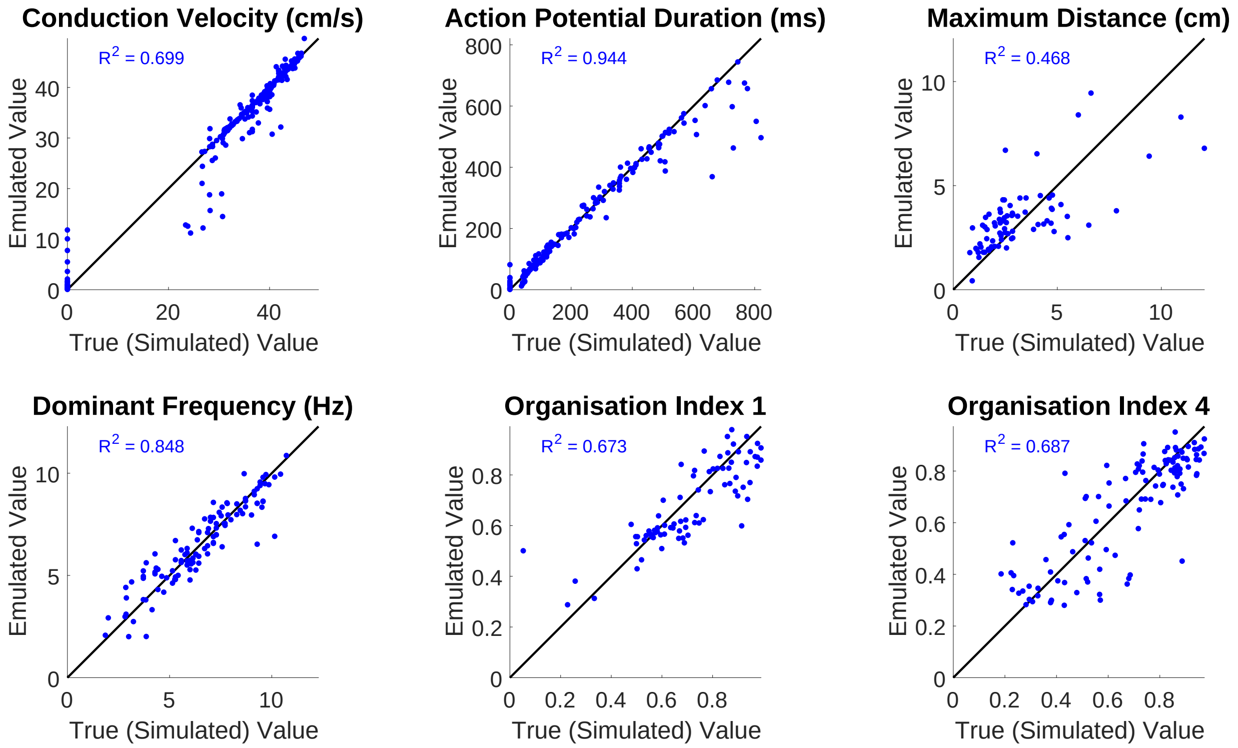

Figure S3. The performance of a partitioned emulator on the eight parameter problem, expressed by comparison between the unseen test data generated by the simulator and the emulator's predictions for those same locations in the parameter space. Only correctly classified and non-null data is visualised and used in R^2 calculation, so classification accuracy must also be taken into account in interpreting these results. The tissue-level biomarkers remain very well emulated in this much higher-dimensional case, as does the dominant frequency. Occasional data points in the other biomarkers show more significant errors, but there does not appear to be a bias to emulation error, suggesting general trends are still captured. Due to the stochastic nature of the hyperparameter optimisation for SVM, 20 samples are used for each amount of training data.

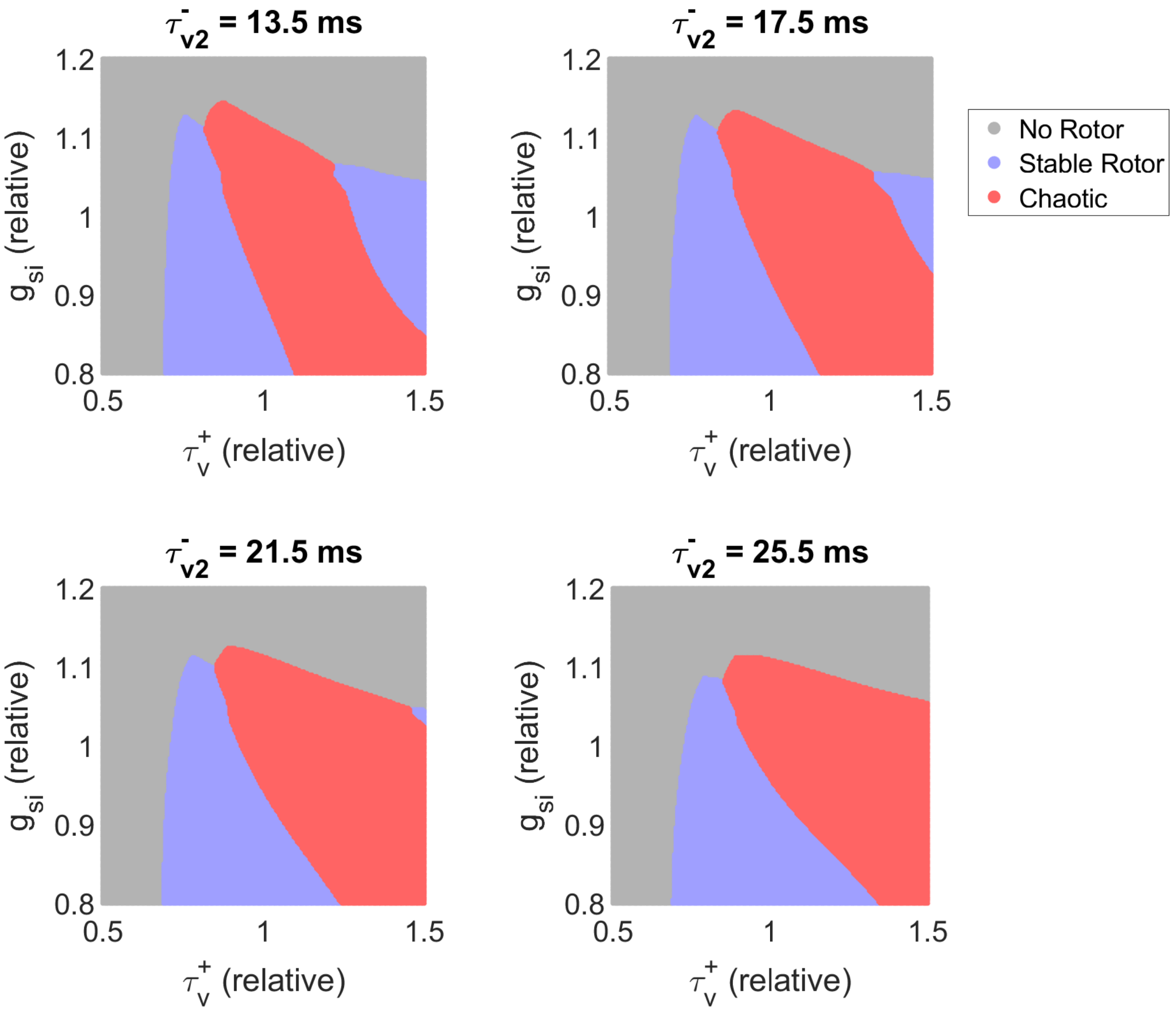

Figure S4. Parameter maps as predicted by the classifier model. Exchanging g_{fi} for τ_v^+ has little effect on how τ_{v2}^- modulates the two parameters of interest (compare with Figure 8 in main document). A critical window of excitability associated with risk of wave breakup is still observed, and responds in the same fashion to slowed recovery of fast inward channels, which is primarily associated with increased risk of re-entry destabilisation.
